# Skin tissue engineering advances in severe burns: review and therapeutic applications

**DOI:** 10.1186/s41038-016-0027-y

**Published:** 2016-02-19

**Authors:** Alvin Wen Choong Chua, Yik Cheong Khoo, Bien Keem Tan, Kok Chai Tan, Chee Liam Foo, Si Jack Chong

**Affiliations:** 1 Singapore General Hospital, Department of Plastic, Reconstructive and Aesthetic Surgery, 20 College Road, Academia Level 4, Singapore, 169845 Singapore; 2 Singapore General Hospital, Skin Bank Unit, Block 4 Level 3 Room 15, Outram Road, Singapore, 169608 Singapore; 3 Transplant Tissue Centre, c/o Skin Bank Unit, Singapore General Hospital, Block 4 Level 3 Room A7, Outram Road, Singapore, 169608 Singapore

**Keywords:** Burns, Skin tissue engineering, Stem cells, Cultured epithelial autografts, Dermal substitutes, Microskin grafting

## Abstract

Current advances in basic stem cell research and tissue engineering augur well for the development of improved cultured skin tissue substitutes: a class of products that is still fraught with limitations for clinical use. Although the ability to grow autologous keratinocytes in-vitro from a small skin biopsy into sheets of stratified epithelium (within 3 to 4 weeks) helped alleviate the problem of insufficient donor site for extensive burn, many burn units still have to grapple with insufficient skin allografts which are used as intermediate wound coverage after burn excision. Alternatives offered by tissue-engineered skin dermal replacements to meet emergency demand have been used fairly successfully. Despite the availability of these commercial products, they all suffer from the same problems of extremely high cost, sub-normal skin microstructure and inconsistent engraftment, especially in full thickness burns. Clinical practice for severe burn treatment has since evolved to incorporate these tissue-engineered skin substitutes, usually as an adjunct to speed up epithelization for wound closure and/or to improve quality of life by improving the functional and cosmetic results long-term. This review seeks to bring the reader through the beginnings of skin tissue engineering, the utilization of some of the key products developed for the treatment of severe burns and the hope of harnessing stem cells to improve on current practice.

## Background

Despite the recent question on whether skin is the largest organ in the human body [1], no one can dispute its protective, perceptive, regulatory and cosmetic functions. The top layer of the skin, the epidermis which comprised mainly of keratinocytes, is critical for survival as it provides the barrier against exogenous substances, chemicals, pathogens and prevents dehydration through the regulation of fluid loss. Other cells within the epidermis include melanocytes which give pigmentation and Langerhans’ cells which provide immune surveillance. Beneath the epidermis, the dermis is a thicker layer of connective tissues that consists mainly of extracellular matrix (ECM) or structural components (predominantly collagen and elastin) which give mechanical strength, elasticity and a vascular plexus for skin nourishment. Cells interspersed within the ECM include fibroblasts, endothelial cells, smooth muscle cells and mast cells [2]. These two morphologically distinct layers — the epidermis and the dermis — are in constant communication across various levels (example at the molecular or cellular level, growth factor exchange, paracrine effects, etc.) to establish, maintain, or restore tissue homeostasis. Between the epidermis and dermis is the basement membrane (BM), a highly specialized ECM structure (composed of a set of distinct glycoproteins and proteoglycans) that physically separates the two layers rendering primarily a stabilizing though still dynamic interface and a diffusion barrier [3]. In general, the BM contains at least one member of the four protein families or subtypes of laminin, type IV collagen, nidogen, and perlecan, a heparan sulfate proteoglycan [4]. Populating the epidermal and dermal layers are the various skin appendages such as the hair follicles, sweat glands, sebaceous glands, blood vessels and nerves.

**Fig. 1:**
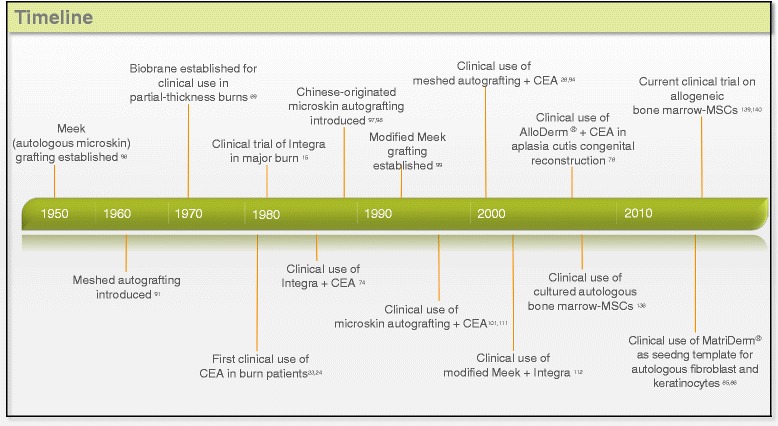
Timeline of skin tissue engineering in burn surgery

Extreme loss of skin function and structure due to injury and illness will result in substantial physiological imbalance and may ultimately lead to major disability or even death. As much as it is claimed that tissue-engineered skin is now a reality to treat severe and extensive burns, the fact remains that current skin substitutes available are still fraught with limitations for clinical use. This is clearly evident amongst burns or wound-care physicians that there is currently no single tissue-engineered substitute which can fully replicate the spilt-thickness skin autografts for permanent coverage of deep dermal or full thickness wounds in a one-step procedure. Indeed, clinical practice for severe burn treatments have since evolved (Fig. 1) to incorporate some of these tissue-engineered skin substitutes (Table 1), usually as an adjunct to speed up epithelisation for wound closure and/or to improve quality of life by improving functional and cosmetic results long-term. However, we must not lose hope, relook at our current practices, press on with innovation and develop new strategies in biology, material science and technological know-how as we seek to achieve the holy grail of creating a fully functional tissue-engineered composite skin with appendages for the clinics.

**Table 1:**
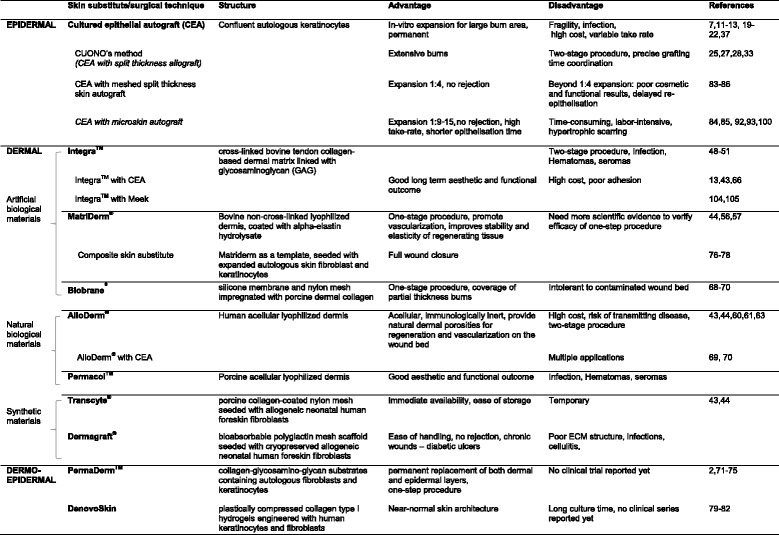
Tissue-engineered skin substitutes and current surgical techniques

## Review

### Birth of skin tissue engineering

#### A coincidence?

The year 1975 seems to be a special year for skin tissue engineering, even before the term “tissue engineering” was officially adopted more than a decade later by the Washington National Science Foundation bioengineering panel meeting in 1987 [5] and later its definition elucidated further by Langer and Vacanti [6] in 1993. The beginnings of skin tissue engineering can be attributed to the pioneering work of two groups in the United States forty years ago. First, Rheinwald and Green reported the successful serial cultivation of human epidermal keratinocytes in vitro [7] in 1975 and later made possible the expansion of these cells into multiple epithelia suitable for grafting [8] from a small skin biopsy. In today’s term, the work is termed “tissue engineering of the skin epidermis”. Concurrently, Yannas, Burke and colleagues reported their maiden work on the in vitro and in vivo characterization of collagen degradation rate [9] in 1975 which we believe pave the way for the design of artificial biological dermal substitute [10], resulting in the “tissue engineering of the skin dermis”.

#### Another coincidence?

**Fig. 2:**
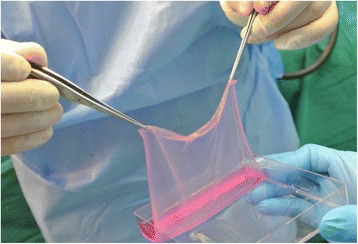
Cultured epithelial autograft supported on a fibrin mat [38] used at the Singapore General Hospital Burns Centre to treat major burns

Interestingly, six years later in 1981, both groups independently reported the clinical use of their respective tissue-engineered substitutes for the treatment of severe and extensive burns, albeit in different approaches. O’Connor et al. reported the world’s first grafting of extensive burns with sheets of cultured epithelium (expanded from autologous epidermal cells) on two adult patients with success at the Peter Bent Brigham Hospital [11, 12]. These autologous cultured sheets (Fig. 2) termed cultured epidermal autografts (CEA) were also subsequently demonstrated to provide permanent coverage of extensive full thickness burns in another two paediatric patients [13].

Meanwhile, Burke et al. (a few months after O’Connor et al.’s report) reported the successful use of a physiologically acceptable artificial dermis in the treatment of extensive burn injuries with full thickness component on ten patients [14]. This was followed by a randomized clinical trial for major burns led by Heimbach et al. [15] on the use of this artificial dermis, now known as Integra^TM^ Dermal Regeneration Template. This successful multi-centre study involving eleven centres and many other studies [16, 17] might have inevitably given this dermal substitute a “gold standard” status for full thickness burns treatment [18].

While ground breaking, the work of the above two groups are still far from reaching the ultimate goal of replacing skin autografts for permanent coverage of deep dermal or full thickness wounds in extensive burns.

### CEA: a bumpy ride for prevalence in the clinics

#### Importance of Cuono’s method

**Fig. 3:**
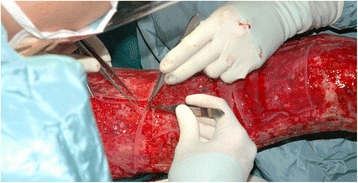
Grafting of cultured epithelial autografts on allodermis at Singapore General Hospital Burns Centre based on Cuono’s two-stage method

One of the main disadvantages of the CEA technology was apparently the lack of consistency in engraftment, with poor “take” reported mainly on wounds devoid of dermal elements, even with properly cultured keratinocytes [19–22]. It was later demonstrated in the mid-1980s by Cuono and his colleagues on the importance of having the dermal component present when they reported good graft take of the CEA laid on healthy vascularized allogeneic dermis in a full thickness wound bed [23, 24]. For the Cuono’s method to be effective, a two-stage procedure is required. First, there must be available human skin allografts ready to be grafted on excised full thickness wound. This is followed by a wait of about two to three weeks which would provide the patient with necessary protection and coverage as the underlying cadaver dermis vascularizes while the autologous epithelial sheets from the harvested small skin biopsy can be prepared simultaneously by culture. When the cultures are ready, the highly immunogenic cadaver epidermis placed on the patient earlier will have to be removed by dermabrasion to make way for the CEA to be grafted (Fig. 3). This two-stage composite allodermis/cultured autograft technique has been adopted by several centres with fairly reproducible success since the 1990s [25–27]. One relatively recent success story came from the Indiana University experience that reported a final graft take of 72.7 % with a 91 % overall survival rate on eighty-eight severe burn patients. These results as the authors mentioned “gives much optimism for continuing to use CEA in critically burned patient” [28].

#### The detractors

**Fig. 4:**
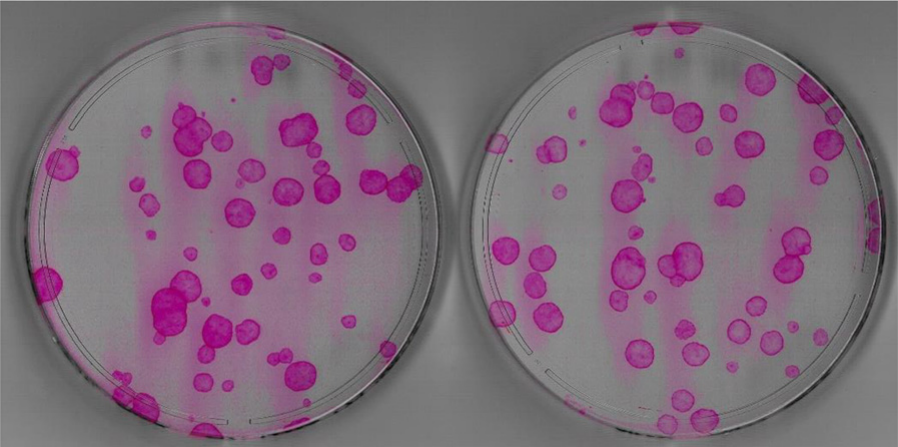
Colony forming efficiency assay: a simple way of measuring the clonogenic ability of keratinocytes and estimating the growth capacity of these cells

However, there are still detractors to this Cuono’s method for a number of reasons. Firstly, there might not be readily available skin allografts, especially in the East Asian region where organ and tissue donation is still not prevalent [29, 30]. In addition, skin allografts carry some risks of infection and antigen exposure [31]. Secondly, the timing of the CEA placement could be a tricky balancing act. It was mentioned that if cadaver skin or epithelium is rejected or sloughed off prior to the availability of cultured epidermal grafts for the burn patients, the opportunity to use the cadaver dermis as vascularized dermal support (based on Cuono’s method) might be lost [32]. The coordination of CEA use with the timing of surgery is therefore a concern. In another scenario, the wound bed might be ready for CEA grafting but yet the cultured keratinocytes were not ready or sufficient for grafting. On the other hand, there were situations where the CEA cultures were ready for grafting but the wound bed was not or the patient was too sick to undergo surgery. It is known that once the keratinocytes form a sheet in culture, the sheets need to be used within the shortest time as possible to maintain efficacy especially for treatment of full thickness burns [28, 33]. Otherwise, the keratinocyte stem cell population in the cultures would be compromised and these critical cells for regeneration would move towards an irreversible unidirectional process from holoclones (stem cells) to paraclones (highly differentiated cells) [34–36]. In such a case, the efficacy of the CEA would drop drastically, rendering poor engraftment and sub-optimal wound healing [37]. Even though there was a recommendation to use colony forming efficiency assay of keratinocytes (Fig. 4) as an indirect and simple quality check for the “regenerative property” of CEA cultures [36, 38], there were not too many adopters.

CEA sheets are fragile in nature and extreme care must be taken to avoid tangential and shearing forces while moving the patient’s limb or repositioning the patient to prevent any loss of the cell layers. Therefore not surprising, it was reported that CEAs placed on anterior sites were amendable to improved take rates [28]. However with the need to keep the grafted site completely immobile [39] and given the limited sites for grafting of CEAs (recommended to be placed on “non-pressure sites” to prevent shearing off of these friable grafts), these led to some form of resistance to CEA use by certain burn surgeons. In addition, the higher vulnerability of CEA to bacterial contamination on the wound site which could result in almost complete loss of the grafts compared to meshed autograft [22, 40] also exacerbate the reluctance of CEA use in the clinical setting.

#### Issue of cost

Finally, the high cost of production of CEA has often been quoted as one of the major hindrance for its widespread use in many review papers [37, 39, 41]. This cost is going to escalate further as there is a trend of directing cellular therapeutic products with “substantial manipulation” (this would include keratinocyte expansion) to be produced in a Good Manufacturing Practice (GMP) setting for administrative demands like quality, safety controls and regulations [42]. GMP is a pharmaceutical quality system which ensures that products are consistently produced in a tightly-controlled cleanroom environment according to stringent quality standards. Typically, adoption of this practice especially for autologous human cellular therapeutic products would entail much higher cost in terms of overheads such as manpower and facility resources as there is no economy of scale for such tailored cellular products unlike the manufacturing of allogeneic cells [43].

### Dermal substitutes: a not so bumpy ride for prevalence in the clinics

#### Two-stage procedure

Based on the knowledge that there are now many dermal substitute products available commercially and with many of such products widely reviewed and tested in both pre-clinical and clinical settings [2, 18, 32, 41, 43–46], it is self-evident that the challenges for their therapeutic use (especially for acellular ones) is less than CEA (cellular-autologous products) insofar as their respective functional requirements (dermal versus epidermal) are totally different. If epidermis is “life”: providing the protection crucial for our survival, then dermis is the “quality of life”. Most current biocompatible dermal substitutes are to a certain extent able to mimic the basic properties of the ECM in the human skin by providing some form of structural integrity, elasticity and a vascular bed. However, the fact remains that these products lack an epithelial layer and in most cases, the use of such products will need to be followed up with grafting of split thickness skin autograft for permanent coverage, usually in a two-stage procedure. While there are advantages of harvesting thinner split-thickness skin autografts and that donor sites heal faster [15], there is still harvest site morbidity with a possibility of insufficient donor sites in extensive burns.

#### Integra^TM^

Being the most widely accepted artificial biological dermal substitute [47], the use of Integra^TM^ which is made up of bovine collagen and chondroitin 6-sulfate, has been reported to give good aesthetic and functional outcomes when compared to using split thickness skin autograft alone [48]. However, it is known that infection still remains the most commonly reported complication of Integra^TM^ [49–51] . Meticulous wound bed preparation before the use of this template (or similar type of artificial biological materials) has been reported to be critical to ensure good take. Otherwise with the collection of hematomas and seromas beneath the material, the product is susceptible to infection resulting in a costly loss of an expensive tissue-engineered product and manpower time, while increasing the length of hospital stay for the patient.

But with much progress in the development of newer wound care products, the use of advanced antimicrobial silver dressing such as Acticoat dressing as an overlay to Integra^TM^ [44] as well as the use of topical negative pressure or vacuum assisted closure (VAC) in combination with Integra^TM^ [52–54] have been reported to mitigate the rates of infection with positive results. In one study, it was reported that the application of topical negative pressure dressings to dermal templates can reduce shearing forces, restrict seroma and haematoma formation, simplify wound care and improve patient tolerance; even as it was reported that the negative pressure did not accelerate vascularization of the Integra dermal template based on histological assessment [55].

#### MatriDerm®

Another newer generation of artificial biological dermal substitute that is gaining wider acceptance for use in the clinics recently is MatriDerm®. Made up of bovine collagen and an elastin hydrolysate, this product is touted for use in a single-stage procedure. MatriDerm® was shown to be able to accommodate split thickness skin autograft safely in one step with no compromise in take on burn injuries [56, 57]; and it seemed to be feasible for use in critically ill patients [58]. It was suggested that unlike Integra^TM^ which has antigenic properties due to the presence of chondroitin-6-sulfate, the combination of collagen and elastin in MatriDerm® can promote vascularization quicker through the support of in-growth cells and vessels while improving stability and elasticity of regenerating tissue [44]. Furthermore, higher rate of degradation and difference in neodermal thickness of MatriDerm® compared to Integra^TM^ [59] might give the former an extra edge; even though there is still relatively weak scientific evidence on their comparison in the current literature [58].

#### Other dermal substitutes

There are also other categories of dermal substitutes available commercially. On top of substitutes made from “*Artificial Biological Materials*” described above for Integra^TM^ and MatriDerm®, the other two commonly recognised classifications are : “*Natural Biological Materials*” and “*Synthetic Materials*” [43, 44]. Decellularized human skin allografts (such as AlloDerm®) and decellularized porcine xenografts (such as Permacol^TM^) are dermal products derived from “*Natural Biological Materials*” as typically these products are “de-epidermalized” and processed to remove the antigenic cellular components while retaining the structure of the native dermis. Known as acellular dermal matrix (ADM), the advantage of using this class of product is that the templates derived from decellularized tissues provide natural dermal porosities for regeneration and vascularisation on the wound bed in-vivo. In vitro studies have shown that such products support adhesion, growth, and function of several cell types [60, 61]. In addition, there is partial conservation of BM which might aid epidermal cell attachment [62]. Nevertheless these products are known for their high cost with the risk of transmitting infectious diseases and they are usually used in two surgical procedures [63]. But with advancement in processing of human skin allografts and also with the use of negative pressure therapy, studies using a one-stage procedure of co-grafting with human ADM (CG derm) and autologous split thickness skin grafts have been reported with some success [64, 65].

Finally, dermal substitutes using synthetic materials seem to be less widely used since their inception in the 1990s for burn treatment. Such products include Transcyte®, a porcine collagen-coated nylon mesh seeded with allogeneic neonatal human foreskin fibroblasts bonded to a silicon membrane; and Dermagraft®, a bioabsorbable polyglactin mesh scaffold seeded with cryopreserved allogeneic neonatal human foreskin fibroblasts. It was reported that both of these products are currently off the market but their technologies have been licensed to Advanced BioHealing for further production and marketing to improve the product [44].

This brings to the issue about cost of dermal substitutes. In general, dermal substitutes are deemed to be costly for clinical usage as mentioned in a report comparing the clinical outcome of MatriDerm® and Integra^TM^ [66]. Based on a tabulated comparison of cost per cm^2^ between different dermal substitutes in 2007, it was noted that Dermagraft^TM^ was about twice the cost of Integra^TM^ [67], and that might explain why Dermagraft^TM^ is presently off-market.

#### Biobrane®

**Fig. 5:**
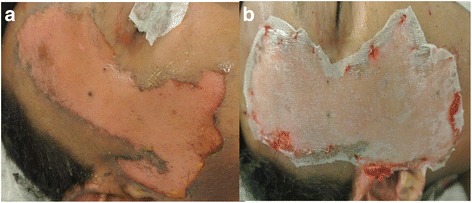
Application of Biobrane. **a.** Before application **b.** After application

As opposed to Transcyte®, Biobrane® is still widely used as a synthetic skin substitute as it is known for its success in the definitive management of partial thickness burns (Fig. 5) in many centres [68–70]. Biobrane® is the exact product of Transcyte® less the neonatal human fibroblasts and is also used as a dressing to hold meshed autografts and cultured keratinocyte suspension [69, 71]. On top of the versatility in usage, the popularity of Biobrane® is likely due to its lower cost and yet, it is as efficacious in treating partial thickness burns compared to Transcyte® [72]. In a recent comparison of Biobrane® and cadaveric allograft for temporizing the acute burn wound, Austin et al. concluded that Biobrane® is superior in terms of lower procedural time and associated cost largely due to the relative ease of application of this product [73]. Indeed, Greenwood et al. in a sharing of their experience using Biobrane® on 703 patients concluded that Biobrane® is relatively inexpensive, easy to store, apply and fix, and reliable when used according to guidelines [69].

Currently, there is also an increasing trend to use Biobrane® as an alternative to cadaver allografts as temporizing dressings after excision of major burn injuries [68, 69, 73]. However, the caveat of using this technique is that the wound bed must be meticulously prepared to prevent any infection and there is still the lack of existing literature and published clinical protocols [68] to prove that it can be a worthy replacement of the human skin allografts, especially in the treatment of full thickness burn wounds.

### Towards a composite skin substitute for permanent replacement

#### Combining CEA and Integra^TM^

The first thing that comes to mind for an autologous composite skin to be used for permanent coverage is to just individually combine the artificial dermal substitute (Integra^TM^) and the CEA on the wound bed. After all, both have their roots in 1975 and their first respective independent clinical use to treat severe burns was reported in 1981. The first hint of their combined use was in 1984 when Gallico et al. reported the permanent coverage of large burn wounds with autologous cultured epithelium in *The New England Journal of Medicine* [13]. In the study, it was mentioned that *Patient 1* with flame burns of 97 % total body surface area had received excision to the level of muscle fascia on certain part of the body and were covered temporarily by human cadaver skin allograft or a collagen-glycoaminoglycans-silastic sheet (later known as Integra). This was followed by grafting with CEA even though it was not mentioned whether the Integra^TM^ was replaced with the cultured epithelium. It was only in 1998 that the use of cultured autologous keratinocytes with Integra in resurfacing of acute burns was presented in a case report by Pandya et al. [74]. Used as a two-step procedure, the authors resurfaced the neodermis (vascularized Integra^TM^) by the third week with ultra-thin meshed autografts and CEA on the anterior torso of the patient in two mirror-image halves. It was found that the CEA performed as well as the side covered with split thickness autograft in terms of appearance, durability and speed of healing. This positive result was not surprising as a month earlier in the same journal, another group [31] reported that vascularized collagen-glycoaminoglycan matrices produced a favourable substrate for cultured epithelial autografts in a porcine model.

Interestingly, there were practically no subsequent bigger clinical series which describe the two-stage use of Integra^TM^ followed by the grafting of CEA. One of the reasons as alluded by Pandya et al. [74] was that of cost when they mentioned the combination of Integra^TM^ and autologous cultured keratinocytes was very expensive. The other reason quoted was that direct application of cultured keratinocytes to an Integra^TM^ wound bed was found to be problematic due to the poor adhesion of the cells to the template [43]. This might be attributed to the lack of fibroblasts migrated into the Integra^TM^ which delayed the maturation of the BM between the epithelial grafts and the neodermis. In a bilayered skin equivalent tested in-vitro, the presence of fibroblasts with keratinocytes was reported to be important for the formation of high levels of collagen type IV and laminin, some of the key elements of the BM [32, 75]. In fact it was further validated later in another skin equivalent model that only in the presence of fibroblasts or of various growth factors, laminin 5 and laminin 10/11, nidogen, uncein, type IV and type VII collagen (all of which are components of the BM) were decorating the dermal/epidermal junction [76].

#### Combining CEA and other skin substitutes

Similarly it was also observed that there were scanty clinical reports on the two-stage use of AlloDerm®, (a decellularized human ADM product that was first approved by the FDA to treat burns in 1992 [77]) and CEA. One notable case report in 2009 was the successful treatment of aplasia cutis congenita using the combination of first applying on the defect with AlloDerm® followed by CEA grafting two weeks later. It was reported that during a two-year follow-up period, there were no complications such as motion limits resulting from hypertrophic scarring or scar contracture. Coincidentally, there was also an earlier attempt in 2000 to use allogeneic dermis and CEA as a one-stage procedure to reconstruct aplasia cutis congenita of the trunk in a newborn infant [78]. While the results were reported to be promising, it was noted that three additional applications of CEAs were required for 90 % of the wound to be healed.

#### Autologous dermo-epidermal composite skin substitutes

By far, the most promising autologous dermo-epidermal (composite) skin substitute reported is the cultured skin substitutes (CSS) developed in Cincinnati in the United States. This substitute is composed of collagen-glycosaminoglycan substrates which contains autologous fibroblasts and keratinocytes. Reported to be able to provide permanent replacement of both dermal and epidermal layers in a single grafting procedure [2, 79–83], this product was later commercialised as PermaDerm^TM^ [43]. PermaDerm^TM^ can currently be engineered within 30 days. It is indicated for the treatment of large full-thickness skin defects, however it has not yet obtained Food and Drug Administration (FDA) approval and clinical trials on its efficacy remain to be seen. More recently, a German group reported the development of an engraftable tissue-cultured composite skin autograft using MatriDerm® as a template for the seeding of expanded autologous skin fibroblasts and keratinocytes [84]. They reported that this developed skin composite has strong homology to healthy human skin based on the characterization of the epidermal strata, comparison of the differentiation and proliferation markers and the presence of a functional basal lamina. This skin substitute was subsequently used clinically on two patients with full thickness wounds. While the wounds are relatively small in size (the largest being 9 x 6 cm), there was positive outcome with full wound closure for all the defects treated [85, 86].

There are many promising autologous cellular bilayered skin substitutes proposed out there such as DenovoSkin developed at Tissue Biology Research Unit, University Children’s Hospital, Zurich, Switzerland. This product is based on plastically compressed collagen type I hydrogels engineered with human keratinocytes and fibroblasts from a small skin biopsy [87, 88]. The same group has further reported for the first time, a more advanced bioengineered human dermo-epidermal skin graft containing functional dermal blood and lymphatic vessels using human keratinocytes,fibroblasts, and microvascular endothelial cells [89, 90]. However the challenge for the utilization of such products remains; that is: how soon can we culture sufficient autologous cells, impregnate them into the scaffold and get the substitute ready for grafting. Time is of essence especially for a massive burn case with little donor site and options.

### Adapting the use of skin tissue engineering products to current practice in the clinics

#### Combining CEA and widely-meshed autografting

**Fig. 6:**
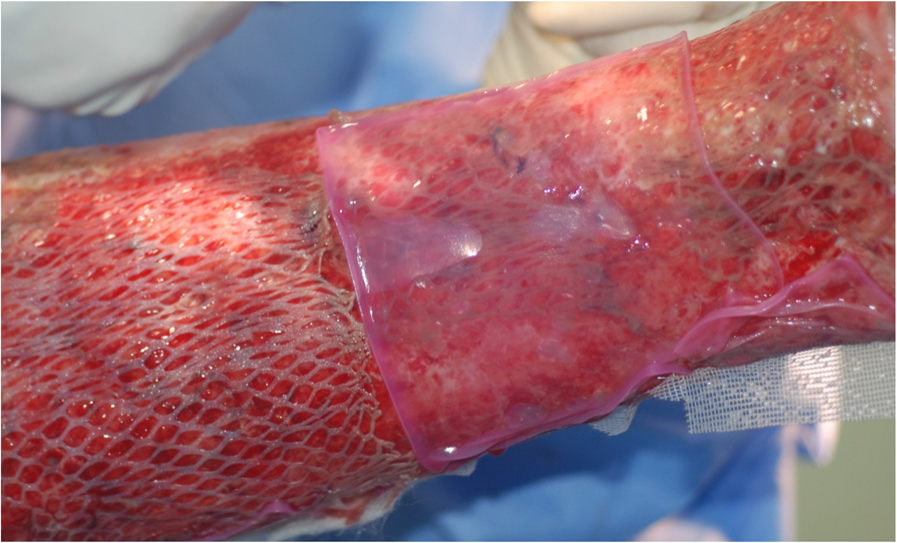
Combining cultured epithelial autografts and widely-meshed autografts

One of the solutions adopted in the clinical setting autografting to quickly treat extensive full-thickness burn wounds is to use widely-meshed split thickness skin grafts to cover the large injured surfaces after the technique of meshing was introduced by Tanner et al. in 1964 [91]. However at expansion rate greater than 1:4, such meshed grafts have been reported to be difficult to handle. Worse still, re-epithelialization might be delayed or even absent when a meshed piece of skin was expanded beyond a ratio of 1:6 [92]; and with substantial areas left uncovered in the interstices, there would be cosmetically unsatisfactory “string vest” appearance [93]. To address these disadvantages, use of CEA in combination with widely meshed autografts (Fig. 6) has been reported with success in a clinical series of 12 children with major burns. As the authors in the study mentioned, this synergistic combination of autografts and autologous cultured epidermis sheets appeared more effective than one of these techniques applied alone [94]. Based on the Indiana University experience of eighty-eight patients who received CEA (an earlier-mentioned study deemed to be one of the success stories in CEA usage), the authors also reported that if an insufficient amount of cadaver dermis remains after allografting (Cuono’s method), 1:6 meshed split thickness autografts (if available) would be placed onto recipient wound bed under the CEA sheets. This was to minimize shear forces and hasten graft take in areas with inadequate allodermis [28]. Other variant technique involving the use of sprayed cultured autologous keratinocytes in combination with meshed autografts to accelerate wound closure in difficult-to-heal burn patients was also reported [95].

#### Resurgence of microskin autografting

**Fig. 7:**
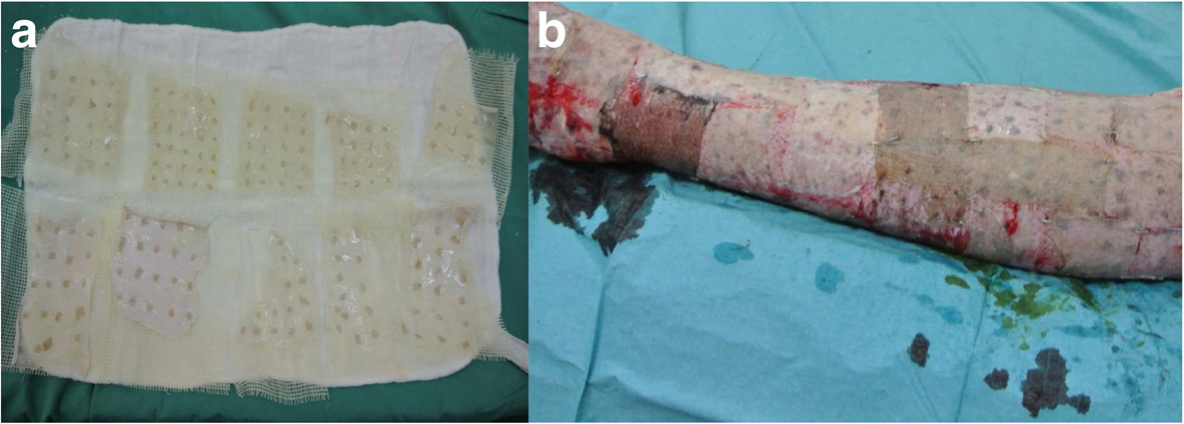
Microskin autografting on an extensive-burn patient at the Singapore General Hospital Burns Centre. **a.** Split thickness skin autografts were cut into small pieces and laid in close proximity with one another on cadaveric allografts. **b.** Sheets of autologous microskin-allografts were grafted onto recipient wound bed

Based on the current literature, there seems to be a resurgent towards the use of autologous microskin grafting (Fig. 7) even though the concept of using small skin bits for autografting was described by Meek in 1958 [96], before the use of meshed grafts. Chinese-originated microskin autografting was described in the 1980s for the treatment of extensive burns [97, 98]. Later in 1993, Kreis et al. improved on Meek’s original technique [99] and popularised the so-called modified Meek method which was found to be superior to widely-meshed autografts when higher expansion rates (up to 1:9) were used in adult patients with major burns [100]. While the modified Meek method or the Chinese-originated microskin grafting method (expansion rate of up to 1:15) is still time-consuming and laborious with the need for more staff in the operating theatre [101], these problems do not seem to serve as a deterrent because this procedure which can be performed almost immediately is seen as life-saving [102]. Outcome is generally positive with reliable take rate even on difficult wound bed [103], shorter epithelization time [101, 104, 105], less prone to loss due to infection [92, 100] as well as satisfactory functional and aesthetic results [106–108]. Moreover if the Meek graft fail, it was restricted to a partial area without affecting the neighbouring skin islands [103] formed from the epithelial migration from the borders of each of the skin bits. More recently, the use of micrograft transplantation with immediate 100-fold expansion for epidermal regeneration on both healthy and diabetic wounds in porcine models was reported [109]. In the same report, it was mentioned early clinical results confirmed the utility of this technique in a case report of a civilian patient with fifty-four percent total body surface area burn admitted to a U.S. Army military hospital in Iraq and successfully treated with the described micrografting technique [110].

#### Combining CEA and microskin autografting

However, scar contracture and hypertrophic scar formation (as would be seen in cases using widely-meshed autografts) are problems frequently associated with microskin autografting, especially where high expansion ratios are used for the treatment of extensive burns with high percentage of deep dermal or full thickness component [92, 93]. Therefore as what was described earlier for widely-meshed skin autografts, CEA was also reported to be used in combination with microskin autografting to accelerate wound closure [93, 101, 111]. Results reported have been positive with one of the earliest studies by Raff et al. describing that the combination of widely expanded postage stamp split thickness grafts and CEA provided an excellent take rate and durable wound closure within a short time while avoiding the problems associated with engraftment of CEA on fascia [101]. Menon et al. also reported that with the use of sprayed CEA and modified Meek technique, they observed no cases of blistering or scar contracture in those treated sites but unfortunately, the problem of hypertrophic scar remained [93].

#### Modified Meek technique and Integra^TM^

The modified Meek technique in combination with Integra^TM^ dermal template in a two-stage procedure has been reported in extensive burns with some success in a case report involving three patients [112]. As well, radical resection and reconstruction of a giant congenital melanocytic nevus with meek-graft covered Integra was also reported [113]. However, there are very few reports that utilised the above described technique subsequently. On top of cost and issue of infection, it can be speculated that the lack of popularity of this two-stage procedure is that it would incur a delay in utilising the microskin for epithelization which is the main strength of the micrografting technique.

### Where is the next trajectory?

#### Stem cells

Advances in research of adult stem cells and embryonic stem cells offer hope for the therapeutic deficiencies in severe burn treatment using existing skin tissue-engineered products. The therapeutic power of stem cells resides in their clonogenicity and potency [114] and these can be delivered in conjunction with skin composites or by various other methods, including direct application [115]. More recently, there is a burgeoning interest in human induced pluripotent stem cells (hiPSCs) as this Nobel-winning technology pioneered by Shinya Yamanaka and his team [116, 117] enables the reprogramming of adult somatic cells to embryonic-stage cells. hiPSCs technology therefore allows for patient- and disease-specific stem cells to be used for the development of therapeutics, including more advanced products for skin grafting and treatment of cutaneous wounds [115]. However, the recent suspension of the world’s first clinical trial involving hiPSCs to treat age-related macular degeneration continues to raise questions about the safety of this new technology. hiPSCs often acquire mutations with epigenetic and chromosomal changes in culture [118]. Hence, human epidermal and mesenchymal stem cells remain the more promising options for clinical use to treat severe burns, at least in the near term.

#### Enriching for epidermal stem cells

Poor engraftment of CEA even on a properly-prepared vascularised wound bed with dermal element is thought to be due to epidermal stem cell depletion during graft preparation. A solution for this would be to start with a pure population or higher percentage of these stem cells as suggested by Charruyer and Ghadially [119]. Epidermal stem cells can be enriched from the patient’s own skin and a recent study demonstrated that ABCG2, a member of the ATP binding cassette (ABC) transporter family, was a robust stem cell indicator in the human interfollicular keratinocytes that could potentially be used to quickly enrich for keratinocyte stem cells [120]. Mavilio et al. showed that sheets of epithelium grown from autologous holoclones or keratinocyte stem cells (modified genetically) could be used to treat a patient with junctional epidermolysis bullosa [121], demonstrating the power of this graft refinement. The use of enriched population epidermal stem cells for the preparation of cultured grafts for patients offers hope of overcoming several limitations of current skin substitutes as in a suitable microenvironment, keratinocyte stem cells can also form appendages such as hair, epidermis and sebaceous glands [122, 123]. However finding or creating that elusive microenvironment (in vivo or in vitro) - to provide the necessary molecular or cellular signals for the stem cells to regenerate a fully functional skin with all its appendages - remains a challenge.

#### Harnessing allogeneic mesenchymal stem cells

During the past decade, adult tissue-derived MSCs have rapidly moved from in-vitro and animal studies into human trials as a therapeutic modality for a diverse range of clinical applications. MSCs raise great expectations in regenerative medicine, not only because of their multipotent differentiation characteristics, trophic and immunomodulatory effects but also for their extensive sources and biostability when cultured and expanded in vitro [124]. Apart from bone marrow and adipose tissues, human MSCs can also be isolated from a variety of other tissues such as the amniotic membrane [125], umbilical cord [126, 127], cord blood [128] as well as the hair follicle dermal papilla [129] and sheath [130, 131].

MSCs have demonstrated a number of properties in-vitro that can promote tissue repair, including the production of multiple growth factors, cytokines, collagens, and matrix metalloproteinases [132, 133] in addition to the ability to promote migration of other skin cells such as keratinocytes [134]. MSCs have also been reported to enhance wound healing through differentiation and angiogenesis [135]. In the current literature, several clinical cases on the use of cultured autologous bone marrow MSCs for localized and topical treatment of chronic wounds have been reported. Yoshikawa et al. treated twenty patients with various non-healing wounds (i.e., burns, lower extremity ulcers, and decubitus ulcers) using autologous bone marrow–derived mesenchymal stem cells expanded in culture and a dermal replacement with or without autologous skin graft [136]. The authors reported that 18 of the 20 wounds appeared healed completely with the cell-composite graft transfer, and the addition of mesenchymal stem cells facilitated regeneration of the native tissue by histologic examination. For allogeneic MSCs usage, Hanson et al. [137] reported the use allogeneic bone marrow- or adipose-derived, MSCs to treat partial-thickness wounds of Göttingen Minipigs and demonstrated the safety, feasibility and potential efficacy of these MSCs for treatment of wounds.

In our opinion, the immunomodulatory effect of MSCs is key to the immediate utilization of these cells for rapid treatment of severe burns. It is now clear that MSCs modulate both innate and adaptive responses and evidence is now emerging that the local microenvironment is important for the activation or licensing of MSCs to become immunosuppressive [138]. Without this property, there is no way we can harness the regenerative and pro-angiogenic effects of the MSCs in the first place. Thankfully, we can have this off-the-shelf option to use MSCs as an allogeneic source of cells which can be pre-tested for safety and potency before use. And as vascularization of dermal template is crucial for permanent skin graft take - whether in a one-stage or two-stage procedure, the presence of allogeneic MSCs would definitely give that extra edge towards angiogenesis.

It is therefore not surprising to learn that the first worldwide clinical trial which uses allogeneic bone marrow MSCs to treat 10 patients with large severe deep burns is in progress in Argentina. This is done by treating the wound with the application of MSCs through a fibrin-based polymer spray over an acellular dermal biological matrix [139]. The same group, Mansilla et al. has just reported their preliminary experience treating a patient with 60 % total body surface burned with positive results [140]. A search using “allogeneic mesenchymal stem cells for burns” in ClinicalTrials.gov (as at Nov 2015) also revealed that two of such trials have been filed [141] which further reinforce the hypothesis that allogeneic MSCs might have a role in major burn treatment.

## Conclusions

Similar to the what was mentioned that no single treatment can be recommended in the management of diabetic foot ulcers based on the current and emerging therapies [142], there is no particular approach that is definitely superior for the treatment of severe burns. But based on existing technologies and products available for rapid coverage of extensive burns wounds - the use of Biobrane or similar products to cover the partial thickness component whilst the coverage of the deep dermal or full thickness component with skin allografts after excision, followed by a definite closure with autografts (meshed, microskin, CEA or in combination) - seem to be one of the efficacious and cost-effective management approaches. If the quality of life of the patients is to be considered such as to reduce scarring and contractures, tissue-engineered dermal templates can be used but they typically come at a cost. Therefore, before technology can catch up in terms of producing a truly functional substitute that comes at a reasonable cost, the need for skin allograft tissue banks, whether local or regional, to serve healthcare centres that treat severe burns cannot be overstated. This is especially true in the event of mass casualty [143]. Having a facility that can double up as both a skin allograft bank and an autologous epithelial cell sheet culture laboratory would be a bonus as we seek to train and build up a critical mass of skin tissue engineers, scientists as well as administrators specializing in finance, quality assurance and regulatory affairs. Only by working closely with clinicians to fully appreciate the requirements for the patients, can this specialized pool of personnel innovate, harness emerging technologies, manage cost and navigate through the regulatory minefields for a realistic advancement of this exciting field of skin-based regenerative medicine.

## Abbreviations

ADM: acellular dermal matrix
ATP: ATP binding cassette
BM: basement membrane
CEA: cultured epithelial autografts
CSS: cultured skin substitutes
ECM: extracellular matrix
FDA: Food and Drug Administration
GMP: Good Manufacturing Practice
hiPSCs: human induced pluripotent stem cells
MSCs: mesenchymal stem cells
VAC: vacuum assisted closure
